# High-Temporal-Resolution High-Spatial-Resolution Spaceborne SAR Based on Continuously Varying PRF

**DOI:** 10.3390/s17081700

**Published:** 2017-07-25

**Authors:** Zhirong Men, Pengbo Wang, Chunsheng Li, Jie Chen, Wei Liu, Yue Fang

**Affiliations:** 1 School of Electronic and Information Engineering, Beihang University, Beijing 100191, China; menzhirong@buaa.edu.cn (Z.M.); wangpb7966@buaa.edu.cn (P.W.); lics@buaa.edu.cn (C.L.); fangyue@buaa.edu.cn (Y.F.); 2 Collaborative Innovation Center of Geospatial Technology, Wuhan 430079, China; 3 Electronic and Electronic Engineering Department, University of Sheffield, Sheffield S1-3JD, UK; w.liu@sheffield.ac.uk

**Keywords:** high-temporal-resolution, high-spatial-resolution, high-squint-angle, synthetic aperture radar (SAR), continuously varying PRF (CVPRF), high-order imaging algorithm

## Abstract

Synthetic Aperture Radar (SAR) is a well-established and powerful imaging technique for acquiring high-spatial-resolution images of the Earth’s surface. With the development of beam steering techniques, sliding spotlight and staring spotlight modes have been employed to support high-spatial-resolution applications. In addition to this strengthened high-spatial-resolution and wide-swath capability, high-temporal-resolution (short repeat-observation interval) represents a key capability for numerous applications. However, conventional SAR systems are limited in that the same patch can only be illuminated for several seconds within a single pass. This paper considers a novel high-squint-angle system intended to acquire high-spatial-resolution spaceborne SAR images with repeat-observation intervals varying from tens of seconds to several minutes within a single pass. However, an exponentially increased range cell migration would arise and lead to a conflict between the receive window and ‘blind ranges’. An efficient data acquisition technique for high-temporal-resolution, high-spatial-resolution and high-squint-angle spaceborne SAR, in which the pulse repetition frequency (PRF) is continuously varied according to the changing slant range, is presented in this paper. This technique allows echo data to remain in the receive window instead of conflicting with the transmitted pulse or nadir echo. Considering the precision of hardware, a compromise and practical strategy is also proposed. Furthermore, a detailed performance analysis of range ambiguities is provided with respect to parameters of TerraSAR-X. For strong point-like targets, the range ambiguity of this technique would be better than that of uniform PRF technique. For this innovative technique, a resampling strategy and modified imaging algorithm have been developed to handle the non-uniformly sampled echo data. Simulations are performed to validate the efficiency of the proposed technique and the associated imaging algorithm.

## 1. Introduction

The Synthetic Aperture Radar (SAR) is a well-established and efficient imaging technique for acquiring high-spatial-resolution (generally called high-resolution), wide-swath images of the Earth’s surface due to its all-time and all-weather imaging ability. Since the first civilian spaceborne SAR, Seasat, was launched in 1978 [1], significant progress has been made in this area. As listed in Table 1, with the launch of the SAR satellites Radarsat-2, TerraSAR-X, TanDEM-X, Sentinel-1a and ALOS-2, the resolution of spaceborne SAR has been upgraded from tens of meters to the meter region [2,3,4,5,6,7,8,9,10]. By employing beam steering techniques, TerraSAR-X Next Generation (TerraSAR-X NG) system will achieve a resolution of up to 0.25 meters, thereby being capable of identifying vehicles or objects of a similar size [11].

In addition to the strengthened high-resolution and wide-swath capability, high-temporal-resolution (short repeat-observation interval) would be another key capability for numerous applications [12]. It is necessary to point out that repeat-observation interval means the temporal gap between acquisitions of two images of the same area. Currently, on-orbit SAR systems can provide products with repeat-observation intervals varying from hours to a day, over a day to weeks by employing satellite constellations or left-and-right looking operation. For example, the images obtained by Radarsat-1/2 have been used for monitoring seasonally or permanently ice-covered ocean regions in the Arctic Ocean [13]. Moreover, the COSMO-SkyMed constellation has provided a sequence of 7 stripmap images with one day repeat-observation interval to monitor the flooding of the Severn and Avon rivers [14].

Moreira and his colleagues from the German Aerospace Center (DLR) have noted that users request time series of high-resolution radar images that are acquired using repetition intervals that are as short as possible to study dynamic processes on the Earth’s surface [15]. Additionally, in 2001, Madsen and Edelstein of Jet Propulsion Laboratory (JPL) proposed that the fine temporal sampling or time series of rapidly evolving phenomena would be essential for disaster management for, e.g., flooding, fires, landslides, hurricanes, and earthquakes [12]. Hence, products with high-temporal-resolution are predicted to play a significant role in urgent applications, although current spaceborne systems are proving to be limited in regard to their acquisition capability. The TerraSAR-X and TanDEM-X constellation, with an along-track separation of approximately 3 s, has demonstrated the feasibility of using multiple platforms for the purpose of short repeat-observation interval products, regardless of economic costs [16]. Moreover, geosynchronous SAR would be another potential technique due to its short repeat period.

In general, the acquisition capability of SAR systems is limited based on their maximum squint angle (here, the squint angle is defined as the deviation from the broadside). Hence, we propose a novel high-squint-angle system intended to acquire short repeat-observation interval and high-resolution Spaceborne SAR images with a single pass and a single platform. In addition to applications with high-temporal-resolution, which will mainly benefit from the strengthened squint illumination capability, large area applications will also be supported:

**Comprehensive information analysis of targets**, based on the higher resolution, separate angle and shorter repeat-observation interval images provided by high-squint SAR systems, will enable more precise recognition and identification.

**Higher quality SAR images**, with reduced effects from speckle, which can be reduced through the use of multi-look images, can be obtained because high-resolution and short repeat-observation interval images can reduce speckle efficiently and without a loss of resolution.

Two key factors should be considered in the proposed concept. The first factor is a high-resolution implementation mode, which would be achieved through illumination of the same ground patch for the duration of the coherent time. To satisfy the demands for high-resolution SAR in many civilian applications, the sliding spotlight mode and the staring spotlight mode have been employed in TerraSAR-X. By focusing the antenna beam to a fixed point, the synthetic aperture length for every scattering point is increased, resulting in a higher resolution.

**Figure 1:**
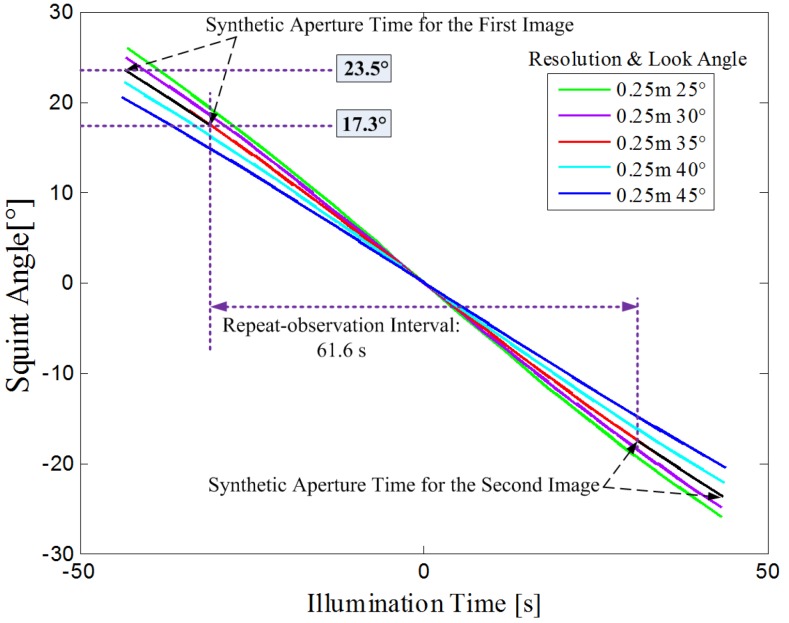
Squint angle as a function of illumination time for different look angles. The illumination time is defined as the time relative to the moment of the minimum slant range. The required squint angle is 23.5${}^{\circ }$ to ensure one minute repeat-observation interval images with a 0.25 m resolution and 5 km swath in both azimuth and range, when the look angle is chosen as 35.0${}^{\circ }$ (red line). Black line represents the synthetic aperture process for images.

Secondly, repeat-observation intervals varying from tens of seconds to several minutes require high squint illumination capability. Consider a planar antenna system similar to TerraSAR-X, as specified in Table 2. The squint angle as a function of illumination time for different look angles is represented in Figure 1, where the required squint angle for one minute repeat-observation interval increases along with a decreasing look angles. For instance, when the look angle is chosen as 35.0${}^{\circ }$, the first image would be achieved with squint angle between 23.5${}^{\circ }$ and 17.3${}^{\circ }$, and the second image would be achieved with squint angle between −17.3${}^{\circ }$ and −23.5${}^{\circ }$. Squint angle from 17.3${}^{\circ }$ to −17.3${}^{\circ }$ is the gap for the repeat-observation interval of 61.6 s.

**Figure 2:**
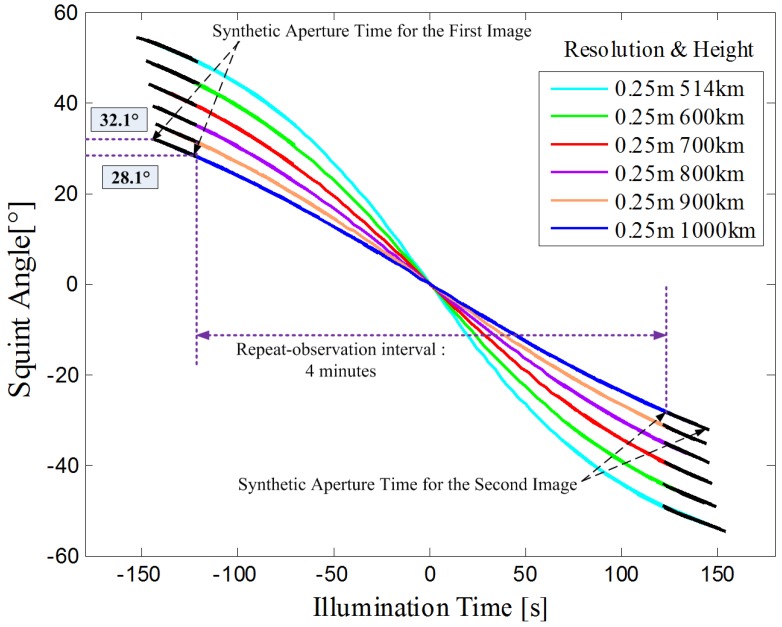
Squint angle as a function of illumination time for different orbit height. Black line represents the synthetic aperture process for images. The look angles of different simulations are chosen as 35${}^{\circ }$. The required squint angle decreases along with an increasing orbit height. For systems with an orbit height of 1000 km, images with repeat-observation intervals varying from tens of seconds to 4 min will be available when the squint angle is higher than 32.1${}^{\circ }$.

The squint angle as a function of illumination time for different orbit height is also represented in Figure 2, where the required squint angle decreases along with an increasing orbit height. For instance, with an orbit height of 1000 km, images with repeat-observation intervals varying from tens of seconds to 4 min will be available when the squint angle is higher than 32.1${}^{\circ }$.

In general, the maximum of repeat-observation intervals mainly depends on orbit height and squint angle. Without loss of generality, we take one minute repeat-observation interval as an example to clarify the proposed technique, and the following simulations are based on parameters in Table 2.

**Figure 3:**
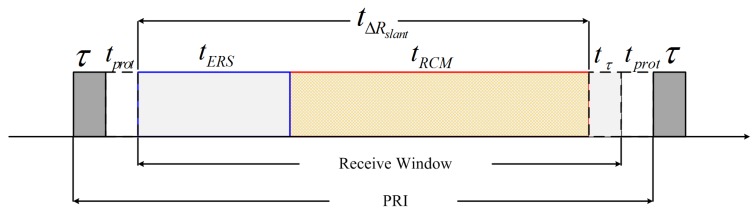
Illustration of the receive window. The PRI (1/PRF) includes a transmitted pulse duration τ, two guard intervals $t_{prot}$, and the receive window. $t_{ERS}$ is the echo time of the effective range swath, $t_{\tau }=\tau$ is the received pulse duration for each point, ensuring that the targets to be imaged are with full range resolution, and $t_{RCM}$ is the duration caused by the RCM. The duration $t_{\Delta R_{slant}}$, related to the span of the slant range, consists of $t_{ERS}$ and $t_{RCM}$.

For SAR systems, high squint angle would cause an increased range cell migration (RCM), which leads to a contradiction between the receive window and ‘blind ranges’ (e.g., nadir echo blockage and transmit pulse blockage). Compared with the conventional stripmap SAR, the RCM of the sliding spotlight and staring spotlight modes at high squint angles takes a substantially greater percentage of the receive window (Figure 3), thereby resulting in a narrow imagery swath.

**Figure 4:**
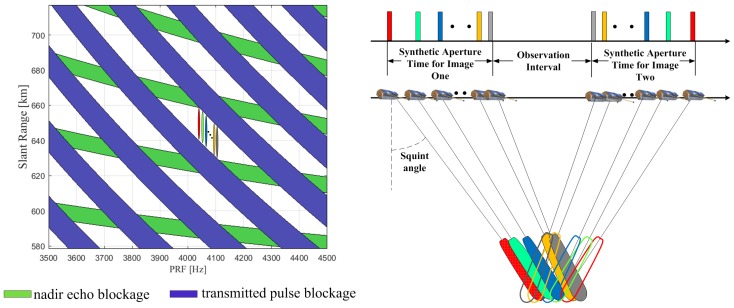
Timing diagram (slant range versus PRF) (**left**) and basic geometry (**right**) of CVPRF conception. Pulses of different squint angle is represented with different color (varying from red to gray). For transmitted pulse on the right of the figure, its suitable PRF is shown on the left with the same color.

The concept of continuously varying PRF (CVPRF) is proposed to overcome this limitation, as shown in Figure 4. With this technique, the RCM can be efficiently reduced by varying the PRF according to the changing slant range (defined as the range between SAR sensor and target) of each transmitted pulse. As apparent from the right of Figure 4, the slant range decreases along with the decreasing squint angle during the acquisition of the first image. For example, to avoid transmitted pulse blockage and nadir echo blockage, we choose 4030 Hz for the red pulse, according to the timing diagram on the left. For the green pulse, the slant range decreases along with the decreasing squint angle. Under this condition, we need to change the PRF for the green pulse, because 4030 Hz is not suitable any more. Similarly, the suitable PRFs should also be changed for pulses with the other colors. For the second image, the suitable PRFs would vary inversely along with the increasing squint angle. Using this technique, echo data of all pulses can be received by SAR sensor and high-squint-angle SAR images can be achieved.

**Figure 5:**
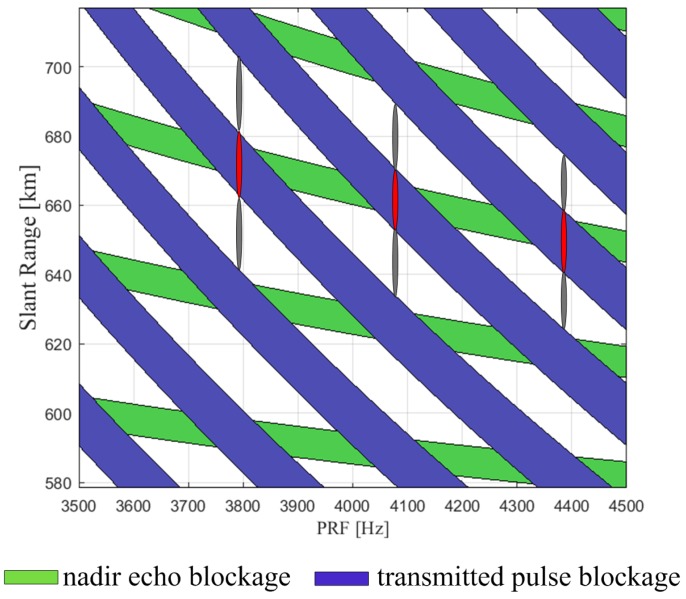
Timing diagram of Staggered-SAR, also called sweep SAR. Both red and gray color represents for beams of Staggered-SAR. The red beam means pulses of the current PRFs is in conflict with transmitted pulse blockage and cannot be received. However, pulses of this slant range still can be received at other PRFs.

Several other continuous PRF variation concepts have been suggested for different purposes. To overcome the limitation of multibeam SAR systems, EADS Astrium and DLR both have proposed Staggered-SAR to image a large continuous swath, as illustrated in Figure 5. According to [17], the strategy of Staggered-SAR attempts to avoid transmitted pulse blockage at the expense of missing pulses for each target, which would lead to a degradation of imaging quality. However, for higher resolution, the missing pulses would increase exponentially, bringing more challenge to recover the missing samples by means of interpolation. The CVPRF technique proposed in this paper, varies the PRF according to the changing slant range, and every transmitted pulse is received efficiently. This technique is effective for high-spatial-resolution and high-squint-angle SAR systems.

This paper is organized as follows: In Section 2, the CVPRF concept is presented in detail, therein showing how the limitation of the RCM is overcome. Moreover, a comparison concerning the data acquisition ability is presented. In Section 3 and Section 4, the design method of the sequence of the PRF is proposed, and range ambiguities are discussed. In Section 5, the relevant imaging algorithm for high-resolution high-squint spaceborne SAR, based on CVPRF, is developed. In Section 6, simulation results are provided, therein showing the accuracy of the resampling processing and the high-order imaging algorithm for the CVPRF concept. Conclusions are drawn in Section 7.

## 2. CVPRF Concept

For spaceborne SAR systems, the antenna length imposes a lower bound on the selected PRF [18]. In return, the PRF limits its ability to continuously acquire echo data in the slant range. As indicated in Figure 3, the span of the slant range $\Delta R_{slant}$, which is related to the length of the receive window, can be expressed as

(1) $$\Delta R_{slant}\leq \left(\frac{1}{PRF}-2\tau -2t_{prot}\right)\cdot \frac{c}{2}$$

where *c* is the speed of light, τ is the transmitted pulse duration, and $t_{prot}$ is the guard interval, which is usually equal to τ.

**Figure 6:**
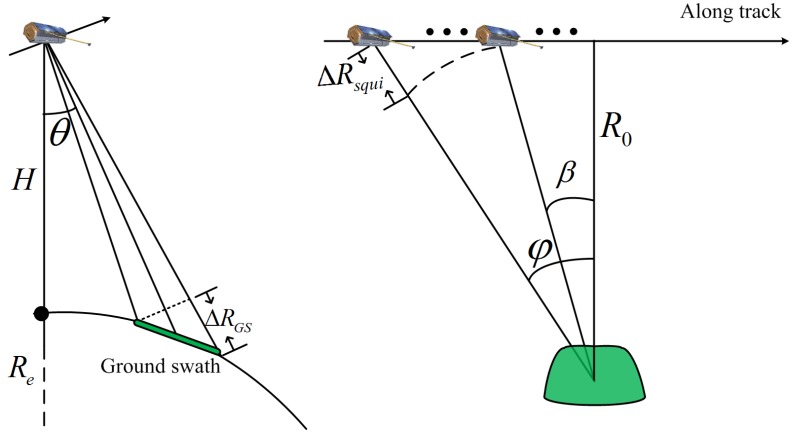
Spaceborne SAR geometry: side-looking geometry (**left**) and along-track geometry (**right**).

The span of the slant range consists of two parts: $\Delta R_{GS}$ (equivalent to $\frac{t_{ERS}\cdot c}{2}$), caused by the ground swath in the range direction, as illustrated on the left of Figure 6, and $\Delta R_{squi}$ (equivalent to $\frac{t_{RCM}\cdot c}{2}$), caused by the squint illumination, as illustrated on the right of Figure 6. The span of the slant range caused by the ground swath can be estimated by

(2) $$\Delta R_{GS}\approx SW\cdot \frac{R_{e}+H}{R_{e}}\cdot \sin\theta$$

where SW is the ground swath, $R_{e}$ is the Earth’s radius, *H* is the orbit height, and θ is the look angle.

For convenience of presentation, the path of the sensor is represented by a linear model. Of course, we need to note that the actual path is substantially more complicated for spaceborne SAR systems. Thus, the span of the slant range caused by the squint illumination can also be estimated by

(3) $$\Delta R_{squi}\approx \frac{R_{0}}{\cos\varphi }-\frac{R_{0}}{\cos\beta }$$

where $R_{0}$ is the slant range at the Doppler center time, φ is the start illumination angle, and β is the end illumination angle.

With Equations ( 2) and ( 3), Equation ( 1) can be expressed as

(4) $$\left(SW\cdot \frac{R_{e}+H}{R_{e}}\cdot \sin\theta \right)+\left(\frac{R_{0}}{\cos\varphi }-\frac{R_{0}}{\cos\beta }\right)\leq \left(\frac{1}{PRF}-2\tau -2t_{prot}\right)\cdot \frac{c}{2}$$

**Figure 7:**
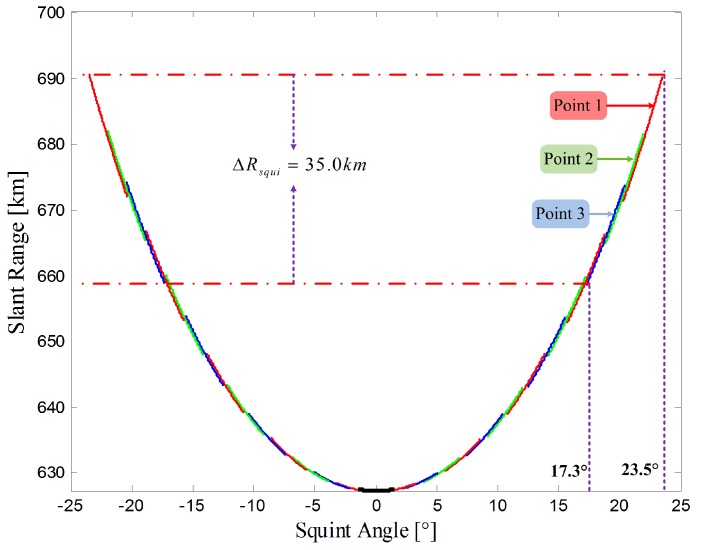
Slant range history of the sliding spotlight SAR. Lines of different color indicate different targets, with a distance of 2.5 km in azimuth, and each line records the slant range history of a target with a 0.25 m resolution; the dark line is the center target of the scene. The distance between Point 1 and Point 3 is 5 km in azimuth; hence, the span of the squint angle for a 5-km azimuth swath is 17.3${}^{\circ }$∼23.5${}^{\circ }$.

With the given orbit height, look angle, PRF and transmitted pulse duration, a compromise between ground swath and azimuth squint angle should be made. Since the antenna beam is always orthogonal to the flight direction for conventional stripmap SAR systems, the span of the slant range caused by squint illumination is negligible. However, for the sliding spotlight mode, apart from the factor of the ground range, the squint illumination angle must be considered. To obtain a more intuitive description of the factor $\Delta R_{squi}$, simulations are performed. The slant range history of the sliding spotlight mode is illustrated in Figure 7. The dark line represents the slant range history of the center target of the scene, similar to the conventional stripmap mode. With increasing squint angle, the spans of the slant range increase almost exponentially. Moreover, the distance between Point 1 and Point 3 is 5 km in azimuth, and the $\Delta R_{squi}$ for a 5 km azimuth swath is 35.0 km.

**Figure 8:**
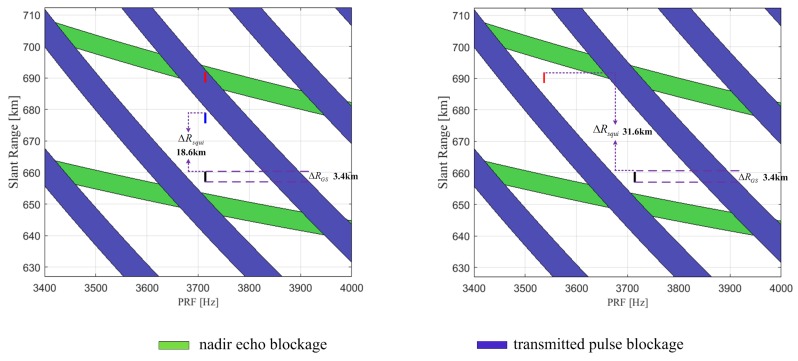
Timing diagrams (slant range versus PRF) of the uniform PRF (**left**) and the CVPRF (**right**). The dark solid line represents the slant range of a 5 km ground swath width with a squint angle of 17.3${}^{\circ }$, and the red solid line represents the slant range of a 5 km ground swath width with a squint angle of 23.5${}^{\circ }$. The slant range of the acceptable squint angle is represented by the blue line, where the maximum of $\Delta R_{squi}$ is limited to 18.6 km.

The comparison of the acquisition capability for a Uniform PRF system and a CVPRF system in the timing diagram would be necessary for a straightforward explanation. We consider a design example with the parameters listed in Table 2, and the simulation results are shown in Figure 8. Here, the dark solid line represents the slant range of a 5 km ground swath width with a squint angle of 17.3${}^{\circ }$, and the red solid line represents the slant range of a 5 km ground swath width with a squint angle of 23.5${}^{\circ }$. For the uniform PRF system, as apparent from the left of Figure 8, the acquisition would be invalid due to the conflict between the echo data and the blind ranges, and the maximum of $\Delta R_{squi}$ is also limited to 18.6 km. However, this limitation would be overcome with the CVPRF system, where the PRF varies with the slant range. As shown on the right of Figure 8, the maximum of $\Delta R_{squi}$ increases to 31.6 km, and the corresponding squint illumination angle can increase up to 23.5${}^{\circ }$.

**Figure 9:**
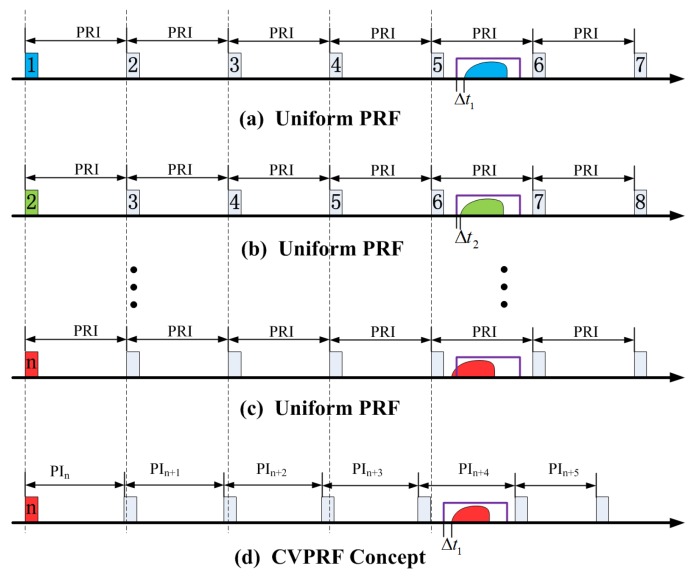
Comparison of the uniform PRF and the CVPRF system with a diagram of the transmitted and received pulses. Different color represents different pulse. The purple box indicates the receive window, and the colored feature in purple box is the echo for its corresponding transmitted pulse. (**a**–**c**) represent the varying position of the echo data for the uniform PRF system. For the sliding spotlight mode, the slant range decreases with decreasing squint illumination angle, and the position of the echo data shifts closer to the beginning of the receive window, eventually running out of the receive window; For the CVPRF system in (**d**), the pulse interval decreases for each new pulse, which is equivalent to a corresponding movement of the receive window, and ensures a proper position for the echo data.

During the acquisition of echo data, the slant range between the SAR sensor and the target varies with the squint angle, which causes a variation in the travel time for different pulses. As we know, for spaceborne SAR systems, the echo of a certain transmitted pulse will be received after several pulses. Without loss of generality, we assume a case in which the reflected pulse is received at the fifth PRI after being transmitted. As shown in Figure 9, the duration between transmitting and receiving can be defined as $\Delta T_{i}=\frac{4}{PRF}+\tau +t_{prot}+\Delta t_{i}$. Here, $\Delta t_{i}$ represents the location of the echo data in the receive window. Given the above-mentioned considerations, the slant range varies with the squint angle, which means that the duration $\Delta T_{i}$ cannot remain fixed, resulting in a changing $\Delta t_{i}$. For certain pulses, the echo data would overlap with the transmitted pulse, called blind ranges (Figure 9c). If, instead of a constant PRF, a sequence of continuously varying pulse intervals, which change according to the slant range, is employed, the interval $\Delta t_{i}$ would be unchanged and would keep the echo away from the blind ranges (Figure 9d). Simulation result has validated the efficiency of the CVPRF technique, allowing echo data to remain in the receive window instead of conflicting with the transmitted pulse or nadir echo.

The comparisons in the timing diagram and in the diagram of the transmitted and received pulses have demonstrated the strengthened acquisition capability of the CVPRF system. Based on the aforementioned analysis, one minute repeat-observation interval images with a 0.25 m resolution and 5 km swath in both the azimuth and range would be practical with parameters similar to those of TerraSAR-X. The following section will analyze and propose an elaborate sequence of PRFs that leads to an efficient echo acquisition for the CVPRF system.

## 3. Design of the Sequence of PRF

As mentioned, the sequence of the continuously varying PRFs is designed according to the history of the slant range to keep the location of the echo data unchanged in the receive window. Consider a case in which the reflected pulse is received at the *M*-th pulse interval (PI) after being transmitted. To facilitate the calculation of $PI_{k+M-2}$, it will be necessary to assume that the pulse intervals from 1 to *M*-1 are equal to $PI_{1}$ at the beginning of the calculation.

**Figure 10:**
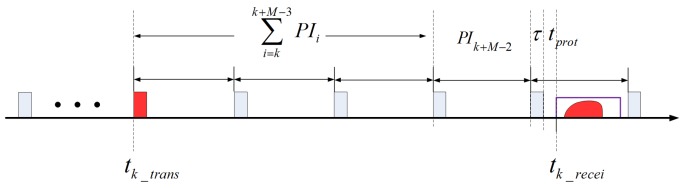
Illustration of the transmit and receive of the *k*-th pulse.

As shown in Figure 10, the *k*-th pulse is transmitted and received at $t_{k\_trans}$ and $t_{k\_recei}$, respectively. $PI_{k+M-2}$ is the unknown variable that we need to solve based on the preceding pulse intervals $PI_{k}∼PI_{k+M-3}$. Then, the transmit time of the *k*-th pulse and the receive time of the *k*-th pulse can be expressed as

(5) $$t_{k\_trans}=\sum_{i=1}^{k-1}PI_{i}$$

(6) $$t_{k\_recei}=t_{k\_trans}+\sum_{i=k}^{k+M-3}PI_{i}+PI_{k+M-2}+\tau +t_{prot}$$

where k_trans denotes the *k*-th transmitted pulse and k_recei denotes the receiving of the *k*-th transmitted pulse. $PI_{k+M-2}$ is the variable that we need to determine.

Using the equation as follows, we can obtain the optimal value of $PI_{k+M-2}$ by an iterative calculation. Both $R(t_{k\_trans})$ and $R(t_{k\_recei})$ can be accurately acquired via a high-precision simulation.

(7) $$\left(t_{k\_recei}-t_{k\_trans}\right)\cdot c=R\left(t_{k\_trans}\right)+R\left(t_{k\_recei}\right)$$

**Figure 11:**
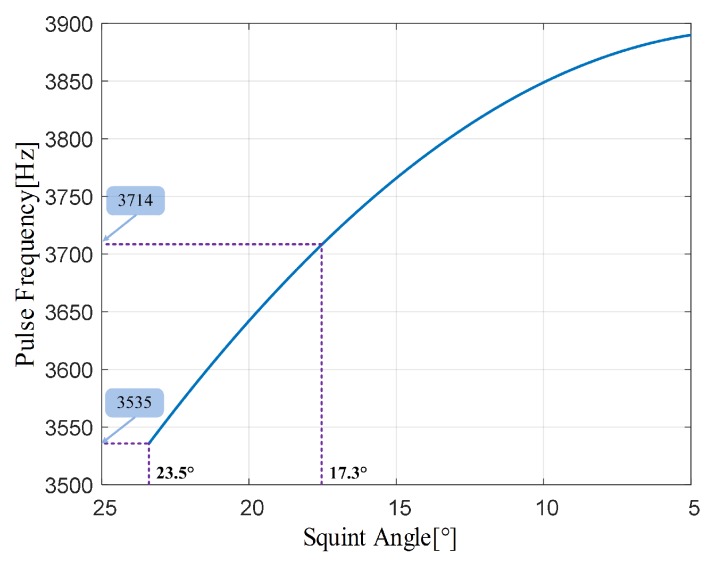
A sequence of a pulse frequency for the CVPRF system. The frequency of the first pulse is 3535 Hz and there are 44,584 pulses during the squint angle changes from 23.5${}^{\circ }$ to 17.3${}^{\circ }$.

Figure 11 shows the result from a simulation performed with the orbit and radar parameters listed in Table 2. The pulse frequency varies from 3535 Hz to 3714 Hz with decreasing squint angle from 23.5${}^{\circ }$ to 17.3${}^{\circ }$.

**Figure 12:**
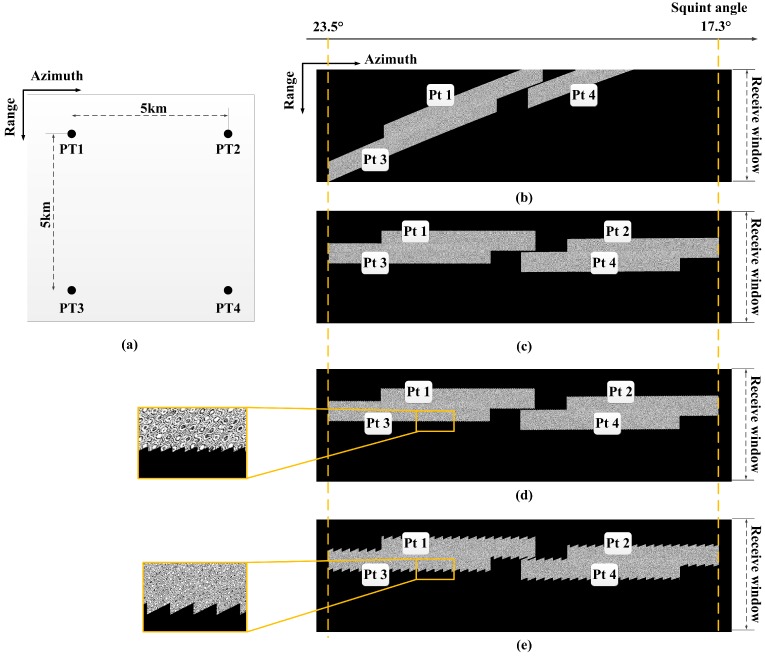
Distribution of targets in the simulation scene (**a**) and the echo data of the uniform PRF (**b**) and CVPRF (**c**); Echo data of CVPRF with the step size of PRF up to 1 Hz (**d**) and 2 Hz (**e**).

To verify the ability to acquire high-resolution and wide-swath SAR images, we study a simulation of the uniform PRF system and the CVPRF system. The simulation scene is shown in Figure 12a, where the distances of the different targets are 5 km in the azimuth and range direction. The echo data of the uniform PRF system and those of the CVPRF system are shown in Figure 12b,c. It can be observed that the echo data of the uniform PRF system cannot be acquired, as the range-walk proliferates at high squint angles. In contrast, the CVPRF system not only can record the echo data of a high squint angle but also has the potential to image larger swaths. The simulation results validate the analysis in Section 2.

Under ideal conditions, the PRFs change continuously. For a compromise and practical strategy, PRFs would change discretely according to the precision of hardware. According to Figure 11, if the step size of PRF is limited to 1 Hz, the PRFs would vary about every 250 pulses and the echo data is shown in Figure 12d. In addition, the echo data with the step size of PRF up to 2 Hz is also shown in Figure 12e.

## 4. Range Ambiguities

**Figure 13:**
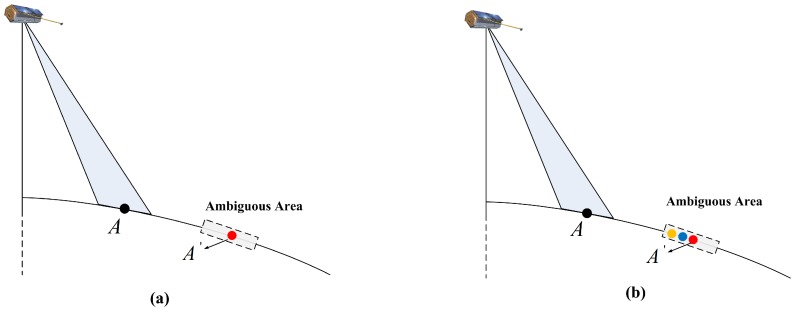
Geometry of the RASR for the uniform PRF (**a**) and the CVPRF (**b**). The red point $A^{\prime }$ is a strong point-like target, and both the blue and yellow points are normal targets.

For traditional SAR systems with a uniform PRF, the position of the ambiguous area is fixed during the aperture time, as shown in Figure 13a. We make an assumption that $A^{\prime }$ is the ambiguous component of point *A*, and it is a strong pointlike target. As a result, after azimuth focusing is applied, the ambiguous energy of point *A* is focused at the same point, making the ambiguity of point *A* much worse than that of other points. In contrast, this situation will be different for SAR systems with changing pulse intervals because the ambiguous components of a strong point-like target are located at different ranges [17]. As illustrated in Figure 13b, the ambiguous position of point *A* shifts from the red point to the blue point and eventually reaches the yellow point. In general, we assume that the red point $A^{\prime }$ is a strong point-like target, and both the blue and yellow points are normal targets. For point *A*, its ambiguous energy exists only in one of the echo pulses instead of in almost all the echo pulses during the aperture time. Moreover, it has to be emphasized that along with the changing PI, the ambiguous energy of the strong point-like target $A^{\prime }$ would spread out to other points, resulting roughly in a uniform distribution of range ambiguity-to-signal ratio (RASR) along the range direction.

**Figure 14:**
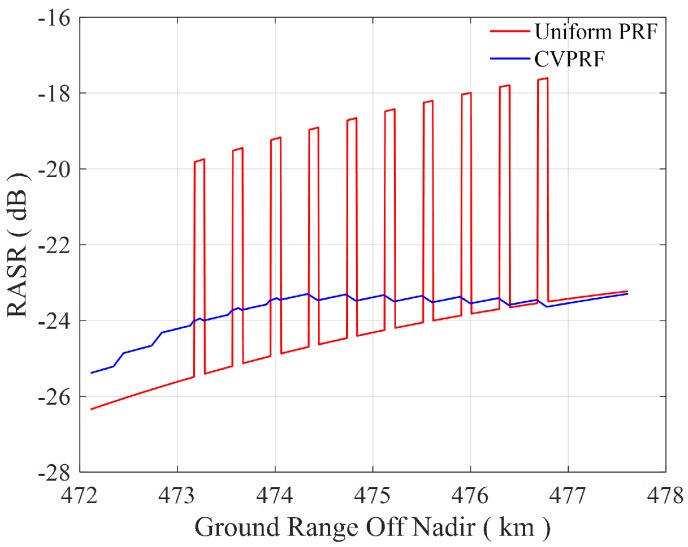
Performance prediction of the RASR for the design example. Ten strong point-like targets are distributed in the ambiguous area.The RASR of uniform PRF system is represented by the red line, and the RASR of CVPRF system is represented by the blue line.

A design example is presented in the following based on the parameters summarized in Table 2. Ten strong point-like targets are distributed in the ambiguous area. In particular, the uniform PRF is chosen as 3714 Hz, and the varying PRFs are chosen in the interval [3535 Hz, 3714 Hz]. The performance prediction of the RASR is shown in Figure 14, where the red line and the blue line represent the RASR of the uniform PRF system and that of the CVPRF system, respectively. Due to ten strong point-like targets distributed in the ambiguous area, the RASR of the uniform PRF system (red line) is above −20 dB, which is regarded as a standard value. By dispersing the ambiguous component to different targets, instead of affecting the same point target, the RASR of all targets will keep under −20 dB, with the help of CVPRF technique. Furthermore, the RASR of the ten targets with strong point-like ambiguous component, is reduced by 4∼6 dB.

## 5. Imaging Algorithm

The key aspect of the imaging process of high-resolution spaceborne SAR is an accurate description of the range history. It has been noted that the range deviation between the actual range history and the equivalent squint range model (ESRM) becomes significant with increasing integration time. To acquire images with an azimuth resolution of 0.25 m, the modified equivalent squint range model (MESRM), which takes the equivalent radar acceleration into consideration, is used in this paper [19]. Since the sequence of pulse intervals is inconsistent, the raw signal recorded by a CVPRF system is inherently nonuniformly sampled. In principle, by focusing each pixel independently in the time domain (e.g., using the BP algorithm), uniform sampling would not be a strict requirement for SAR imaging. However, to reduce the computational cost and apply the conventional SAR processing in the frequency domain, the raw data of the CVPRF system should be resampled to a uniform grid.

### 5.1. Resampling Strategy for the CVPRF System

Based on the MESRM, after demodulation to baseband, the received signal for a point target can be described as follows:

(8) $$s\left(\tau ,t\right)=\sigma_{0}\omega_{a}\left(t-t_{0}\right)\cdot exp\left\{-\frac{j4\pi R(t)}{\lambda }\right\}\cdot \omega_{r}\left(\tau -\frac{2R(t)}{c}\right)\cdot exp\left\{j\pi K_{r}{\left[\tau -\frac{2R(t)}{c}\right]}^{2}\right\}$$

where $\omega_{r}\left(\cdot \right)$ and $\omega_{a}\left(\cdot \right)$ denote the antenna pattern functions in the range and azimuth directions, respectively; $\sigma_{0}$ represents the scattering coefficient; *c* is the speed of light; λ is the signal wavelength; $K_{r}$ is the range chirp rate; τ is the fast time in range; and $t_{0}$ is the Doppler center time.

To compensate for the impact of phase error caused by the sampling time, the received signal should be transformed into the range-frequency and azimuth-time domains using the principle of stationary phases and Fourier transforms. The expression of the echo signal in the range-frequency and azimuth-time domains can be obtained as

(9) $$S\left(f_{\tau },t\right)=\sigma_{0}\omega_{a}\left(t-t_{0}\right)\cdot \omega_{r}\left(-\frac{f_{\tau }}{K_{r}}\right)\cdot exp\left\{-j\pi \frac{f_{\tau }^{2}}{K_{r}}\right\}\cdot exp\left\{-j4\pi R\left(t\right)\left(\frac{f_{\tau }}{c}+\frac{1}{\lambda }\right)\right\}$$

where $f_{\tau }$ is the range frequency.

Due to the non-uniform sampling in the azimuth, the phase error caused by the difference between R(t) and $R(t_{uniform})$ will lead to a failure of the imaging. An effective resampling filter based on phase compensation and Lagrange interpolation has been proposed by [20]. The accuracy of R(t) and $R(t_{uniform})$ is critical to the validity of the resampling. Hence, a geometry simulation is used to obtain R(t), and the MESRM is adopted to calculate the $R(t_{uniform})$. After addressing the non-uniform problem of the CVPRF system, the traditional imaging algorithm for high-resolution wide-swath SAR can be applied.

### 5.2. High-Order Imaging Algorithm for High-Resolution Spaceborne SAR

**Figure 15:**
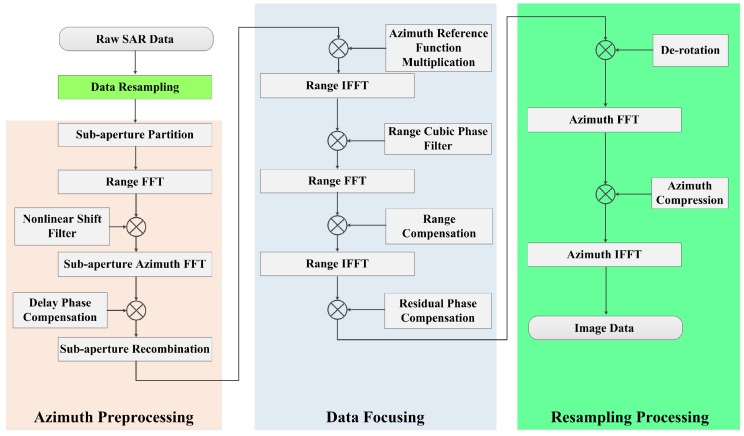
Block diagram of the high-order imaging algorithm.

The block diagram of the proposed high-order imaging algorithm is shown in Figure 15. The imaging process consists of four parts. The first part is data resampling, which is used to transform the non-uniform echo into uniform echo. The second part is azimuth preprocessing, which is performed to remove azimuth aliasing. For high-resolution spaceborne SAR systems, the steering of the antenna beam introduces extra bandwidth. As a result, the insufficient PRF would cause severe azimuth aliasing, especially for a 0.25 m resolution. Using a subaperture partition, nonlinear shift filter, delay phase compensation and sub-aperture recombination, the 2-D signal spectrum data are obtained without aliasing in the azimuth direction. The third part is high-precision focusing within the full swath. With increasing integration time for high-resolution spaceborne SAR, the spatial dependence of the 2-D point scatterer response becomes substantially more significant. By removing the RCM, azimuth modulation, high-order cross-coupling at the reference slant range and range cubic phase filter processing, the coarse focusing within the full swath can be realized. After range compensation and residual phase compensation, the residual RCM and the residual phase error are completely corrected. The last part is resampling processing, which solves the azimuth folding problem in the focused domain. To avoid the azimuth aliasing, an azimuth resampling operation is applied to overcome the constraint on the azimuth swath with a derotation phase function [19].

## 6. Simulation and Results

**Figure 16:**
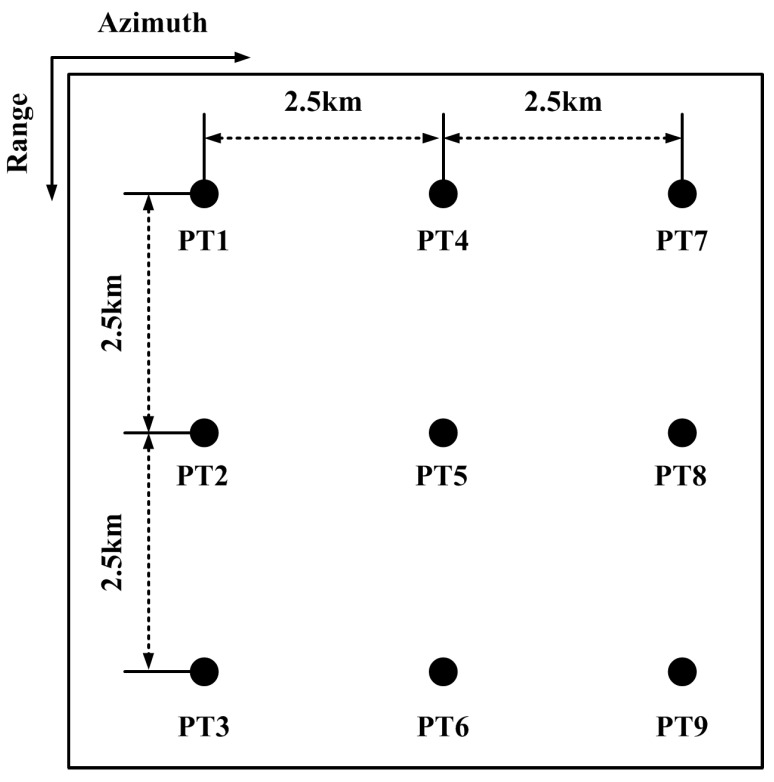
Distribution of targets in the simulation scene.

Here, simulations are performed to verify the accuracy of the resampling processing and the performance of the high-order imaging algorithm. The simulation parameters are listed in Table 2. The simulation scene is shown in Figure 16, where the distances of different targets along the azimuth and range are 2.5 km.

**Figure 17:**
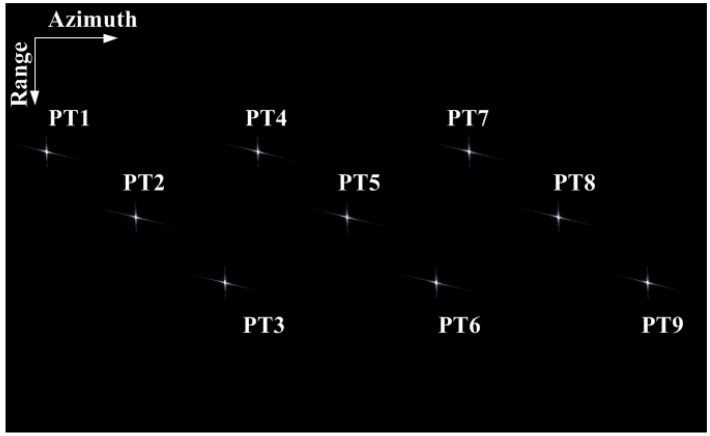
Focused result by the proposed algorithm.

Figure 17 shows that all the targets are well focused after applying our proposed algorithm. To quantify the focusing performance, further results for the point targets are listed in Table 3, using the rectangular window. All these results indicate that the proposed imaging algorithm can effectively satisfy the imaging requirement of a spaceborne SAR system employing the CVPRF concept.

## 7. Conclusions

For high-temporal-resolution and high-spatial-resolution spaceborne SAR, a novel concept based on a sliding spotlight mode called CVPRF has been proposed. This technique offers the potential to overcome the limitation due to conflicts between the receive window and ‘blind ranges’. With parameters similar to TerraSAR-X, one minute repeat-observation interval images with 0.25 m resolution and 5 km swath in both the azimuth and ground range can be achieved by the CVPRF technique. With higher orbit height and higher squint angle, images with repeat-observation intervals varying from tens of seconds to several minutes can be available. Moreover, with the proposed CVPRF technique, large-area applications will also be supported, e.g., comprehensive information analysis of targets and higher quality SAR images. Furthermore, the design of the sequence of the PRF, as well as the performance prediction of the RASR, has been provided. Based on the high-order range equation model, an efficient imaging algorithm for the CVPRF technique has been developed, where resampling processing is introduced to solve the non-uniform sampling problem. In addition, accurate focusing is achieved by a high-order imaging algorithm. Simulation results have been provided to verify the improvement in terms of the effectiveness of echo acquisition and the accuracy of the proposed imaging algorithm. For a compromise and practical strategy, PRFs would change discretely according to the precision of hardware. User requirements always push the development of new technologies. As requirement increases, techniques of varying PRF would become a trend for Spaceborne SAR and difficulties on hardware design would be overcome in the future. 

## Figures and Tables

**Table 1:** Typical SAR systems.

Sensor	Operation	Resolution	Swath	Squint Angle
Seasat	1978	25 m(A) × 25 m(R)	100 km(R)	–
Radarsat-2	2007–Present	3 m(A) × 3 m(R)	20 km(R)	–
TerraSAR-X	2007–Present	1 m(A) × 1 m(R)	5 km(A) × 10 km(R)	2.2${}^{\circ }$
TanDEM-X	2010–Present	1 m(A) × 1 m(R)	5 km(A) × 10 km(R)	2.2${}^{\circ }$
Sentinel-1a	2013–Present	5 m(A) × 5 m(R)	80 km(R)	–
ALOS-2	2014–Present	1 m(A) × 3 m(R)	25 km(A) × 25 km(R)	3.5${}^{\circ }$
TerraSAR-X NG	2018+	0.25 m(A) × 0.25 m(R)	5 km(A) × 5 km(R)	more than 5${}^{\circ }$

**Table 2:** List of simulation parameters.

Parameter	Value	Units
Orbit height	514	km
Eccentricity	0.00018	-
Inclination	97.45	${}^{\circ }$
Longitude of ascend note	92.39	${}^{\circ }$
Argument of perigee	91.28	${}^{\circ }$
Carrier frequency	9.65	GHz
Bandwidth	1.2	GHz
Look angle	35	${}^{\circ }$
Antenna length	4.8	m
Antenna height	0.8	m
Azimuth resolution	0.25	m
Azimuth swath	5.0	km
Range swath	5.0	km

**Table 3:** Performance analysis of point targets.

	Range			Azimuth		
	$\rho_{r}$	**PSLR**	**ISLR**	$\rho_{a}$	**PSLR**	**ISLR**
	**(m)**	**(dB)**	**(dB)**	**(m)**	**(dB)**	**(dB)**
1	0.226	−13.057	−9.649	0.210	−13.254	−10.635
2	0.226	−13.052	−9.646	0.211	−13.256	−10.640
3	0.226	−13.050	−9.647	0.212	−13.260	−10.650
4	0.226	−13.051	−9.656	0.208	−13.258	−10.617
5	0.226	−13.071	−9.638	0.209	−13.256	−10.625
6	0.226	−13.057	−9.652	0.210	−13.254	−10.633
7	0.226	−13.061	−9.656	0.206	−13.254	−10.611
8	0.226	−13.064	−9.657	0.207	−13.256	−10.612
9	0.227	−13.057	−9.656	0.208	−13.254	−10.613
